# Enterococcal Physiology and Antimicrobial Resistance: The Streetlight Just Got a Little Brighter

**DOI:** 10.1128/mBio.03511-20

**Published:** 2021-02-23

**Authors:** Louis B. Rice

**Affiliations:** a Department of Medicine, The Warren Alpert Medical School of Brown University, Providence, Rhode Island, USA

**Keywords:** antimicrobial resistance, carbohydrate metabolism, *Enterococcus*, functional genomics

## Abstract

Enterococcus faecalis differs from many other common human pathogens in its physiology and in its susceptibility to antimicrobial agents. Multiresistant E. faecalis strains owe their phenotypes to a combination of intrinsic and acquired antimicrobial resistance determinants. Acquired resistance is due to E. faecalis frequenting multicultural environments, its capacity to mate with different species, and the nullification of its own defense mechanisms in some lineages. Intrinsic resistance is a complex phenomenon that is intimately tied to the physiology of the species. In their recent study in *mBio*, Gilmore and colleagues (M. S. Gilmore, R. Salamzade, E. Selleck, N. Bryan, et al., mBio 11:e02962-20, 2020, https://doi.org/10.1128/mBio.02962-20) use functional genomics to explore the genetic underpinnings of E. faecalis physiology and antimicrobial resistance. While they do not come up with many definitive answers, their work points the way toward new and fruitful areas of investigation.

## COMMENTARY

Most people are familiar with the old joke in which a policeman sees a drunk man searching for something under a streetlight and asks what the drunk has lost. He says he lost his keys, and they both look under the streetlight together. After a short time, the policeman asks if he is sure he lost them here, and the drunk replies no, that he lost them in the park. The policeman asks then why he is searching here, and the drunk replies, “This is where the light is.” This joke demonstrates what has been termed the “streetlight effect,” where people in many walks of life, including science, tend to look for answers where it is easiest.

That the enterococci (most notably the two species Enterococcus faecalis and Enterococcus faecium) are more resistant to environmental insults than the streptococci to which they are normally compared has been known for decades. Early phenotypic characterizations made note of their resistance to bile and growth in high concentrations of salt. Penicillin susceptibilities made it clear that enterococci differed from streptococci. They were also resistant to killing by single agents but susceptible to killing when β-lactams were combined with an aminoglycoside. The availability of radioactive streptomycin allowed Moellering and Weinberg (1) to suggest that this bactericidal “synergism” was due to the cell wall-active agent facilitating uptake of the aminoglycoside, where its action at the ribosome was lethal.

In the 1980s, DNA homology studies indicated that the enterococci lacked sufficient homology with other streptococcal species to be included in the same genus, and the genus *Enterococcus* was born. Radiolabeled penicillin allowed the discovery that E. faecalis and E. faecium had specific penicillin-binding proteins (PBPs) demonstrating lower affinity for penicillin than others, and lower than any of the PBPs seen in streptococci, providing an early explanation for the reduced susceptibility to β-lactams seen in enterococci. In the 1970s, the ability to follow movement *in vitro* of antimicrobial resistance phenotypes between strains of the same (and sometimes different) species and the emergence of cloning as a routine technique (followed by the advent of PCR in the 1980s) led to the discovery of enterococcal transferable plasmids, conjugative and nonconjugative transposons, and their smaller relatives the insertion sequences. For a time, it seemed that much of acquired enterococcal resistance could be explained by the movement of these individual mobile elements. This pattern continued with the discovery of vancomycin resistance transposons Tn*1546* (VanA) (2) and Tn*5382* (VanB) (3), but then something interesting was observed. Transfer of these individual elements was almost always accompanied by the transfer of large segments of genomic DNA, without any apparent consistency.

Whole-genome sequencing afforded an explosion of genomic data indicating that any order we perceived in the movement of resistance determinants was fanciful. The real story was that many species, including enterococci, have highly plastic genomes that are exchanged with some frequency. The named mobile elements were but a smaller, more readily visible piece of a much larger puzzle. The recent availability of hundreds or even thousands of genomes from a variety of species has revolutionized our understanding of molecular epidemiology, core genomes, and transfer/integration mechanisms and made it clear that any logic we impose on the movement of genetic material is not shared by the species we study.

We soon realized that not all phenomena could be explained by amino acid substitutions encoded by mutated genes. Changes in regulation of single or multiple genes were initially difficult to detect, but broad analysis of transcriptional profiles became possible with the availability of microarrays and transcriptome sequencing (RNA-seq). Epigenetic phenomena such as methylation of DNA can also be important in phenotypic changes. But a fundamental challenge became clear—where do we start looking? Many genes in the enterococcal genome are unannotated, and those that are annotated may not be correct. How do we focus where we look so that we might actually have a chance to find our keys?

In their recent study in *mBio*, Michael Gilmore and colleagues (4) endeavor to move the field of enterococcal physiology and antimicrobial resistance forward by using functional genomics to identify previously uncharacterized genes important for enterococcal fitness, carbohydrate metabolism, and antimicrobial resistance. Gilmore’s laboratory group has a decades-long history of enterococcal investigation, beginning with plasmid and cytolysin analysis, moving forward into pathogenesis, then taking on and defining the complex mechanisms of large-scale genome exchange in E. faecalis (5). Eventually, they turned their interest to the historical evolution of enterococci and have used their prodigious sequencing and analysis capacity to postulate that the “hospital-adapted” enterococci of today likely branched off approximately 425 million years ago, coincident with the animal movement from the sea onto the land (6). This group chooses to tackle big questions and has the brains and wherewithal to do so successfully.

In their ambitious paper in *mBio*, Gilmore and colleagues performed whole-genome *mariner* random mutagenesis of clinical isolate E. faecalis MMH594, a strain isolated from a hospital outbreak that has been archived without passage since 1985. Their goal was to identify previously uncharacterized genes that were involved in enterococcal fitness (defined as growth in Mueller-Hinton broth) and those responsible for intrinsic enterococcal resistance to any of 10 different antibiotics (defined as growth in the presence of 1/8 of the strain MIC). The mathematics required were complex, and a host of exclusions were made to make the work manageable. Only structural genes were considered. The number of insertions in a locus was compared to a pretest estimation of the expected number of insertions based on the number of TA sequences (targets for *mariner* insertion) in the open reading frame (ORF). Only insertions into the middle 80% of the ORF were considered. Mathematical calculations were made to allow for the increased number of insertions expected near the chromosomal origin of replication and the decreased number of insertions expected near the terminus. Decisions were made regarding what level of insertions compared to predicted were considered critical (<1% of expected) or important (<10% of expected). Insertions were normalized for the local environment, and finally, the literature was searched for analogs in related species that had been shown to be important for the specific phenotype.

Once the results were in, further adjustments needed to be made. A substantial percentage of the critical genes were found in phage genomes that were not shared by all enterococci. These were excluded as likely lysogeny-control genes, insertion into which would turn on phage-induced lysis. Based on that same principle, they searched for toxin-antitoxin genes and found several, most also associated with phages. Only one such pair was found to have significantly fewer insertions than expected, but when all were combined (they are on average about half the size of the average gene), insertions into antitoxin genes were statistically significantly less than predicted.

Several interesting findings come out of this work. The first of these is the identification of many critical and important genes related to iron and molybdenum uptake and the incorporation of these metals into proteins. That these genes are important for enterococcal growth is understandable given the need for maintaining redox balance in a species that lacks the typical electron transport chain found in other species. Second, it has been known since the 1950s that E. faecalis has a functional Entner-Doudoroff (ED) shunt off the pentose phosphate pathway that is important for metabolizing glucose and coping with environmental stress (7, 8). The investigators were unable to identify an analog of one of the critical genes in this pathway, ED dehydratase (*edd*), but identified three homologs of the other essential gene ED aldolase (*eda*). They used bioinformatic techniques using the 3 *eda* homologs as bait to create a model to explain the existence of an ED pathway in the absence of an *edd* homolog. This proposed pathway is now ripe for investigation.

The analysis of antimicrobial resistance was internally validated by the appearance of several genes known to be important for resistance to cephalosporins (CroRS, IreK) or daptomycin (LiaRS). It was somewhat disappointing that many of the loci described conferred resistance to polymyxin, a peptide antibiotic that is not used for enterococcal infections. There were some intriguing novel sites relating to resistance to daptomycin, a peptide used to treat enterococcal infections. I find most intriguing a locus (EF 1909) that the authors mention almost as an afterthought. This gene has no attributable function, it is stated to be quite specific to enterococci, and insertion into it sensitizes the strain to all three of the β-lactam antibiotics tested (ampicillin, ceftriaxone, and penicillin). Sounds like a gene well worth investigating.

Like large database studies in clinical medicine, microbiology studies such as this are properly thought of as hypothesis-generating investigations. Rarely will we find definitive answers when we cast such a wide net. But we should come away with more and better questions. Gilmore and colleagues are to be commended for providing us with a better roadmap for future investigations, for giving us leads that will allow anyone in the scientific community who has the interest and expertise to test reasonable hypotheses involving E. faecalis physiology and resistance mechanisms.

Though this study is quite comprehensive, it is important to recognize that it is limited in some ways. Only structural genes were considered. Only the middle 80% of genes was included. One insertion at a time was examined. Arbitrary limits were set for categorizing genes as critical or important. In other words, there is much of the enterococcal genome remaining to be explored. We are still looking under the lamp post. Thanks to this study, the light cast by that lamp just got wider and brighter.
